# *Bacillus* Classification Based on Matrix-Assisted Laser Desorption Ionization Time-of-Flight Mass Spectrometry—Effects of Culture Conditions

**DOI:** 10.1038/s41598-017-15808-5

**Published:** 2017-11-14

**Authors:** Lin-Jie Shu, Yu-Liang Yang

**Affiliations:** Agricultural Biotechnology Research Center, Academia Sinica, Taipei, 11529 Taiwan

## Abstract

Matrix-assisted laser desorption ionization time-of-flight mass spectrometry (MALDI-TOF MS) is a reliable and rapid technique applied widely in the identification and classification of microbes. MALDI-TOF MS has been used to identify many endospore-forming *Bacillus* species; however, endospores affect the identification accuracy when using MALDI-TOF MS because they change the protein composition of samples. Since culture conditions directly influence endospore formation and *Bacillus* growth, in this study we clarified how culture conditions influence the classification of *Bacillus* species by using MALDI-TOF MS. We analyzed members of the *Bacillus subtilis* group and *Bacillus cereus* group using different incubation periods, temperatures and media. Incubation period was found to affect mass spectra due to endospores which were observed mixing with vegetative cells after 24 hours. Culture temperature also resulted in different mass spectra profiles depending on the temperature best suited growth and sporulation. Conversely, the four common media for *Bacillus* incubation, Luria-Bertani agar, nutrient agar, plate count agar and brain-heart infusion agar did not result in any significant differences in mass spectra profiles. Profiles in the range *m/z* 1000–3000 were found to provide additional data to the standard ribosomal peptide/protein region *m/z* 3000–15000 profiles to enable easier differentiation of some highly similar species and the identification of new strains under fresh culture conditions. In summary, control of culture conditions is vital for *Bacillus* identification and classification by MALDI-TOF MS.

## Introduction

The genus *Bacillus* is ubiquitous in the environment, including in terrestrial and aquatic systems, and in the atmosphere. Members of the *Bacillus* genus are rod-shaped, endospore-forming, Gram positive bacteria that widely affect human life and the ecological environment^1^. Over 200 species of *Bacillus* have been identified and differentiated by genetic approaches and also through biological assays according to their activities in the environment or their pathogenicities^2,3^. One method of identifying bacteria is 16S rRNA gene-based taxonomy, but some *Bacillus* species are highly similar according to 16S rRNA sequence^4^. The *Bacillus subtilis* group, *Bacillus cereus* group and *Bacillus pumilus* group have multiple highly-related species within their groups. In the *B. subtilis* group, *B. amyloliquifaciens*, *B. atrophaeus*, *B. licheniformis*, *B. mojavensis*, *B. subtilis* subsp. *spizizenii*, *B. subtilis* subsp. *subtilis* and *B. vallismortis* are included but share different features^5^. The *B. cereus* group comprises *B. anthracis*, *B. cereus*, *B. mycoides*, *B. pseumycoides*, *B*. *thuringiensis* and *B. weihenstephaniensis*, and they are differentiated mainly according to their pathogenicities to humans, insects or others and free-living^2^. The *B. pumilus* group contains *B. altitudinis*, *B. aerophilus*, *B. safiensis*, *B. pumilus* and *B. stratosphaericus* which are differentiated through the *gyrB* (β-subunit of DNA gyrase) sequence and bacterial phenotypic features, chemotaxonomic characteristics and DNA-DNA connectedness^5^. Multi-locus sequence typing and whole genome sequencing^6^ can be used to differentiate close *Bacillus* species and even strains, but no high-throughput approach is commonly applied for classifying and identifying the many *Bacillus* isolates found in environmental samples.

Matrix assisted-laser desorption ionization time-of-flight mass spectrometry (MALDI-TOF MS) has been used for microbial identification and commercialized for more than a decade^7,8^. Several companies along with homemade hardware and software platforms have supported the identification of clinical microorganisms and an abundance of datasets have been compiled^9^. The ribosomal peptides/proteins extracted from microbial samples are the targets of MALDI-TOF MS analysis, and can provide overall and unique spectra of the samples^10^. The mass spectral profiles are compared with microbial protein profile databases through different algorithms which give possible classifications between sample and references. The technique is highly efficient and reliable and results are obtained within an hour^11^.

Identification of *Bacillus* species through MALDI-TOF MS has been conducted for several years and is often applied to important clinical pathogens like *B. anthracis* and *B. cereus* ^12–15^. However, pretreatment for *Bacillus* identification is not as simple as for other bacteria because of endospore formation. Endospores have different protein expression compared to vegetative cells and small acid soluble proteins (SASPs) have a crucial role in endospore formation^16,17^. In anthrax diagnosis, for example, endospore protein composition was shown to differentiate between pathogenic and non-pathogenic *B. cereus* group species^18,19^. If *Bacillus* are classified through general pretreatment of culture, extraction and matrix deposition for MALDI-TOF MS analysis, endospore protein signals cannot be ignored^20^. In addition, incubating *Bacillus* under different conditions influences the protein percentages due to different extents of endospore-formation^21^. Furthermore, different extents of endospore-formation affect the comparison of spectra when *Bacillus* is grown under different culture conditions. Despite culture conditions being critical for accurate identification, a systematic protocol has not been established for the classification of most *Bacillus* species from the environment.

In this study, we evaluated the influence of culture conditions including incubation time, temperature and culture medium and identified the factors that crucially affect accurate classification when *Bacillus* is isolated from different environmental origins. Furthermore, we offer new insights that may strengthen the classification within the *B. cereus* group and the *B. subtilis* group by including different molecular weight regions observed by MALDI-TOF MS.

## Results

### 16S rRNA analysis of *Bacillus* strains

**Figure 1:**
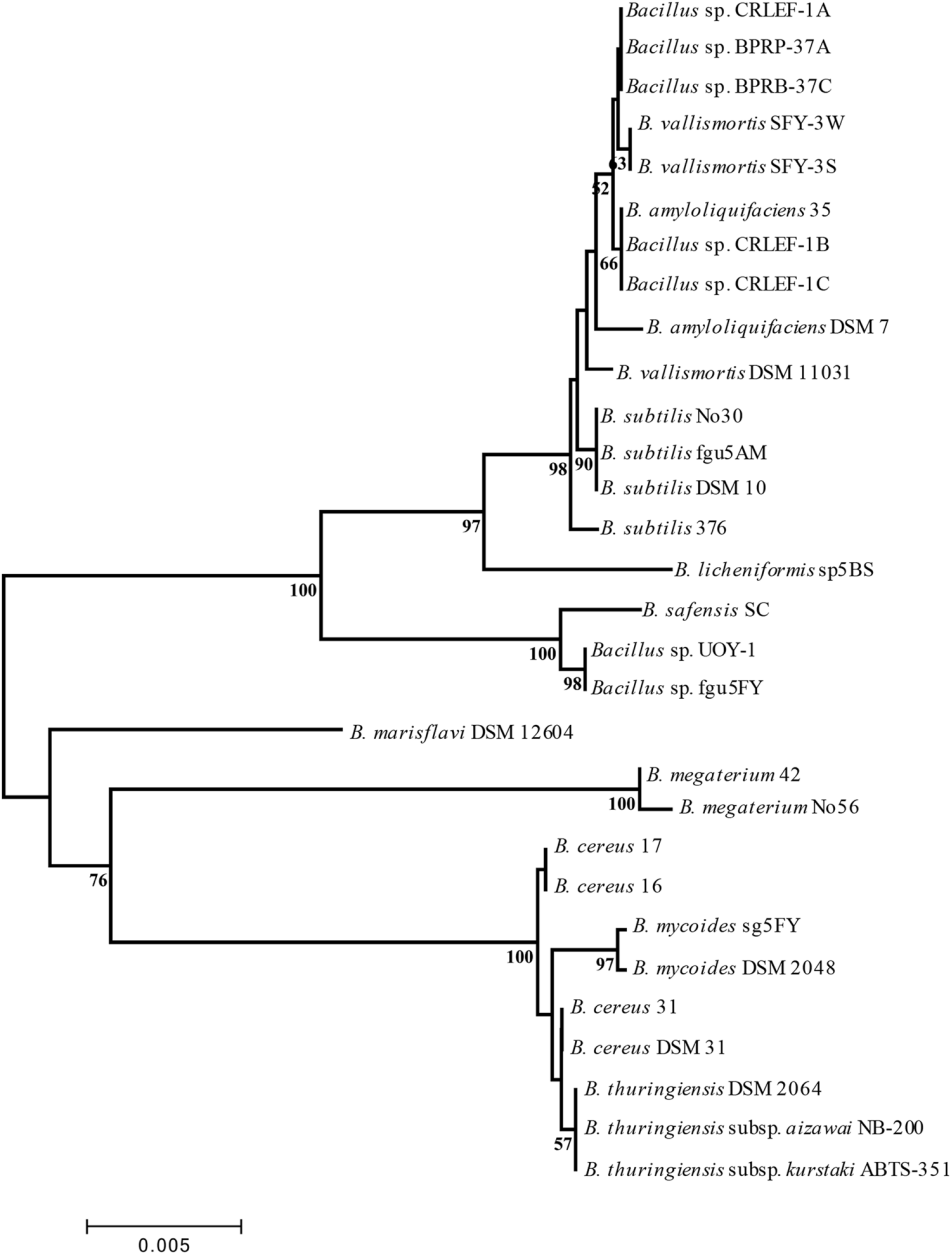
Phylogenic analysis of the *Bacillus* species 16S rRNA sequences using the neighbor-joining method. The sequences were aligned and analyzed using the neighbor-joining method with 1000 bootstrap replications using MEGA 5. The numbers over 50% on the branches represent the bootstrap value and the bar indicates a phylogenetic tree distance of 0.5%.

We sequenced and compared 16S rRNA of 30 *Bacillus* strains which were collected from multiple origins and isolated through different media by ourselves and the Bioresource Collection and Research Center (BCRC) in Taiwan (Table 1). In our collections, the *B. subtilis* group, containing three species, *B. amyloliquefaciens*, *B. subtilis* subsp. *subtilis* and *B. vallimortis*, and the *B. cereus* group including type strains *B. cereus*, *B. mycoides* and *B. thuringiensis* were the two main groups according to the 16S rRNA phylogenetic tree which revealed high similarity within each group of strains (Fig. 1). In each group, the *Bacillus* strains that shared 100% 16S rRNA sequence similarity were grouped together. For example, in Figure S1, the *B. vallimortis* strain SFY-3S and the SFY-3W strain showed entirely different morphology on Luria-Bertani (LB) agar, but could not be differentiated by their 16S rRNA sequences.

**Table 1:** List of *Bacillus* used in this study as well as 16S rRNA accession number, the source of isolation or order, the original cultivation medium, and culture temperature.

Organism	16S rRNA accession Number	Source	Original Medium	Original Culture Temperature
*B. thuringiensis* DSM 2046	D16281	**Type strain, BCRC**	Nutrient agar	30 °C
*B. mycoides* DSM 2048	AB021192	**Type strain, BCRC**	Nutrient agar	25 °C
*B. subtilis* subsp. *subtilis* DSM 10	AJ276351	**Type strain, BCRC**	Nutrient agar	30 °C
*B. amyloliquefaciens* DSM 7	AB006920	**Type strain, BCRC**	Nutrient agar	30 °C
*B. cereus* DSM 31	AE016877	**Type strain, BCRC**	Nutrient agar	30 °C
*B. vallismortis* DSM 11031	AB021198	**Type strain, BCRC**	Nutrient agar	30 °C
*B. marisflavi* DSM 16204	AF483624	**Type strain, BCRC**	Bacto marine broth	30 °C
*B. cereus* 31	KY819017	Terrestrial soil	Potato dextrose agar	25 °C
*B. cereus* 17	KY819023	Terrestrial soil	Brain-heart infusion	37 °C
*B. cereus* 16	KY819024	Terrestrial soil	Brain-heart infusion	37 °C
*B. thuringiensis* subsp. *kurstaki* ABTS-351	KY819035	Commercial biopesticide	Nutrient agar	30 °C
*B. thuringiensis* subsp. *aizawai* NB-200	KY819034	Commercial biopesticide	Nutrient agar	30 °C
*B. mycoides* sg5CY	KY819039	Marine shrimp gut	Yeast malt agar	25 °C
*B. subtilis* 376	KY819033	Terrestrial soil	Luria-Bertani agar	28 °C
*B. subtilis* No30	KY819027	Marine sediment	Luria-Bertani agar	25 °C
*B. subtilis* fgu5AM	KY819036	Fish gut	Marine agar	25 °C
*B. vallismortis* SFY-3W	KY819019	Sea fern	Yeast malt agar	25 °C
*B. vallismortis* SFY-3S	KY819018	Sea fern	Yeast malt agar	25 °C
*B. amyloliquefaciens* 35	KY819021	Terrestrial soil	Potato dextrose agar	37 °C
*Bacillus* sp. CRLEF-1A	KY819028	*Catharanthus roseus* root	Luria-Bertani agar	30 °C
*Bacillus* sp. CRLEF-1B	KY819029	*Catharanthus roseus* root	Luria-Bertani agar	30 °C
*Bacillus* sp. CRLEF-1C	KY819030	*Catharanthus roseus* root	Luria-Bertani agar	30 °C
*Bacillus* sp. BPRB37-A	KY819031	Banana root	Luria-Bertani agar	30 °C
*Bacillus* sp. BPRB37-C	KY819032	Banana root	Luria-Bertani agar	30 °C
*B. licheniformis* sp5BS	KY819038	Sea pig	Yeast malt agar	25 °C
*B. megaterium* No56	KY819025	Marine sediment	Luria-Bertani agar	25 °C
*B. megaterium* 42	KY819022	Terrestrial soil	Potato dextrose agar	30 °C
*Bacillus* sp. fgu5FY	KY819037	Fish gut	Yeast malt agar	25 °C
*B. safensis* SC	KY819026	Bee gut	Luria-Bertani agar	30 °C
*Bacillus* sp. UOY-1	KY819020	Sea urchin	Yeast malt agar	25 °C

### MALDI-TOF MS analysis of *Bacillus* with different incubation temperatures and periods

**Figure 2:**
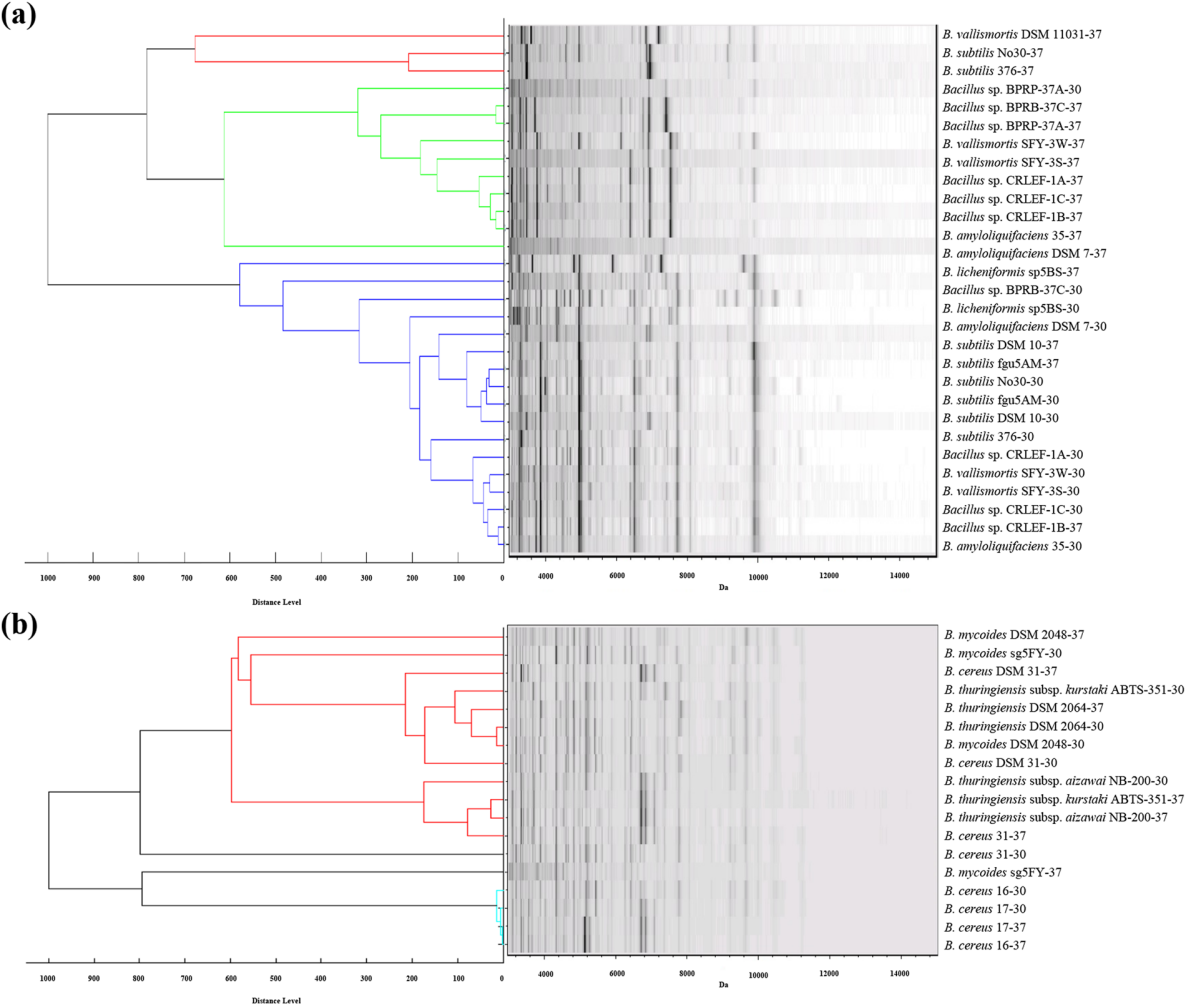
MSP dendrograms and spectra gel view of (**a**) *Bacillus subtilis* and (**b**) *Bacillus cereus* group species incubated at 30 °C and 37 °C for 24 hours. Numbers behind the species and strain name indicate the culture temperature.

The inconsistencies in *Bacillus* identification through MALDI-TOF MS are mostly because of endospore formation and culture conditions. In addition, different species of *Bacillus* isolated from different environments have a variety of optimal growth temperatures which further influences the endospore formation rate. The *B. subtilis* group revealed a consistent main spectrum (MSP) with strains incubated at 30 °C or 37 °C mainly separated into two clusters (Fig. 2a). But the *B. cereus* group did not share similar MSP patterns to the *B. subtilis* group. For example, *B. cereus* 16 and *B. cereus* 17 had similar MSPs at two different temperatures (Fig. 2b).

**Figure 3:**
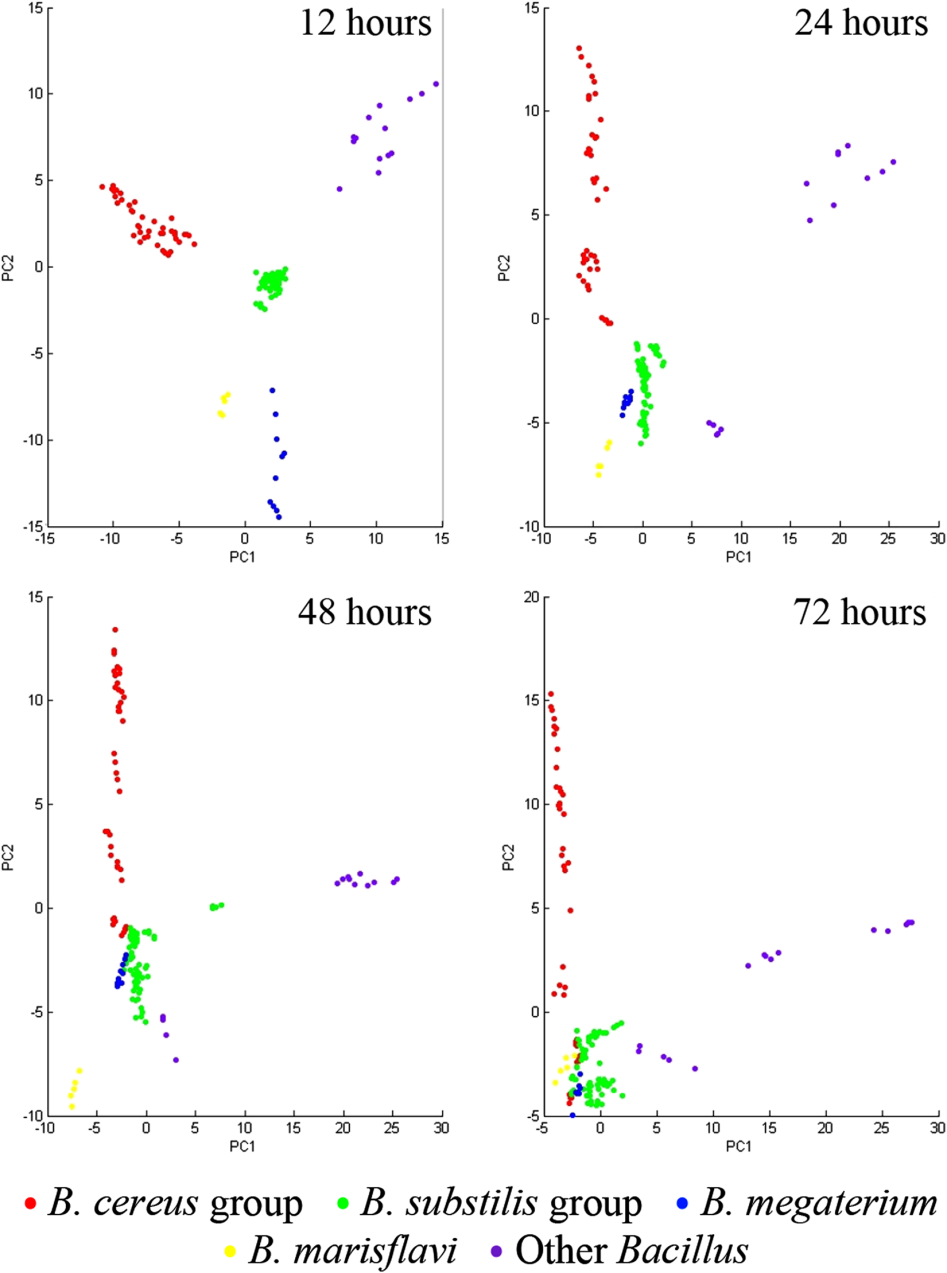
Two-dimensional principal component analysis plots of *Bacillus cereus* group, *Bacillus subtilis* group, *Bacillus megaterium*, *Bacillus marisflavi* and other *Bacillus* species cultured for 12 hours, 24 hours, 48 hours, and 72 hours. The spot presents one spectrum and each *Bacillus* isolate includes five spectra. The plots were generated by first two principal components: PC1 and PC2.

Different incubation times for *Bacillus* ranging from 12 hours to 72 hours had a dramatic effect on the endospore populations (Table S1 and Fig. S2). Principal component analysis plots were used to illustrate the MSP variances in the *Bacillus* groups cultured for different times (Fig. 3 and Fig. S4). At 12 hours, each *Bacillus* group was clearly separated; however, the *B. cereus* group was divided into two sections after incubation for 24 hours. After 48 to 72 hours, several isolates from each *Bacillus* group were not significantly distinguished from other groups in principal component analysis. The MSP dendrograms and spectral gel views are provided in Fig. S3. We then identified SASPs, biomarkers of endospores, and found they were accumulated in both *B. cereus and B. subtilis* samples cultured over 48 and 24 hours, respectively (Fig. S5). These results demonstrated that the uncertainty of endospore formation affects classification using MALDI-TOF MS and fresh *Bacillus* is an ideal sample for classification and identification. But interestingly, the MSPs of a few species such as *B. mycoides* still showed obvious differences between 12 and 72 hours even though almost no endospores were detected within three days incubation (in the period 12–72 hours) (Table S1).

### MALDI-TOF MS analysis of *Bacillus* with different incubation agar media

**Figure 4:**
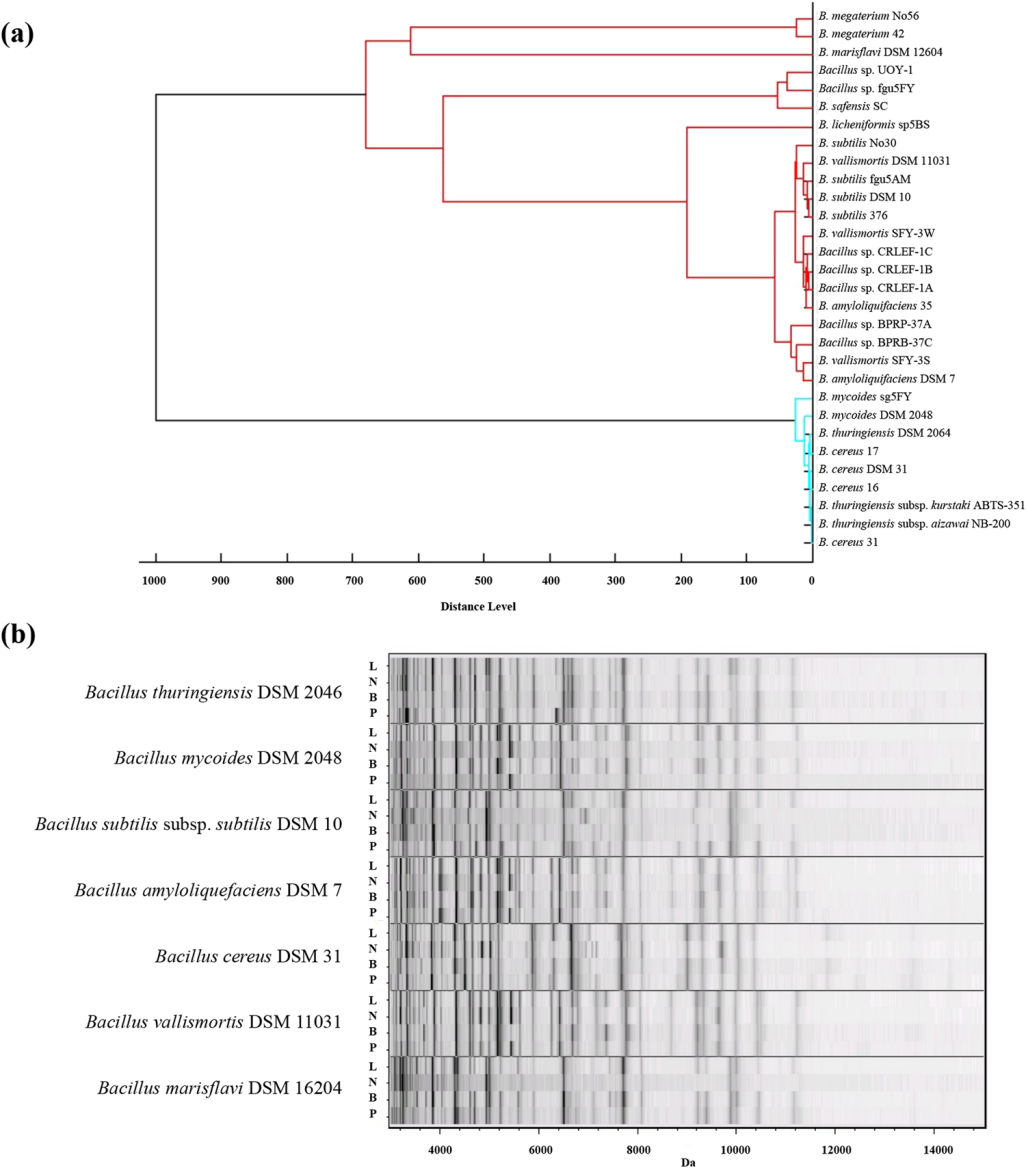
Biotyper analysis of *Bacillus* with different culture media. (**a**) MSP dendrogram of *Bacillus* cultured on LB agar at 30 °C for 12 hours and (**b**) spectral gel view of seven *Bacillus* type strains cultured on LB agar (L), NA (N), BHI agar (B) and PCA (P) at 30 °C for 12 hours.

To further determine the stability and reliability of *Bacillus* classification through MALDI-TOF MS, the samples were cultured and collected 12 hours after incubation and all kept at 30 °C. Then the impact of different agar media was evaluated. LB agar, nutrient agar (NA), plate count agar (PCA) and brain-heart infusion (BHI) agar, which are commonly applied for isolating and culturing *Bacillus* from clinical and environmental samples, were investigated. The four different agar media had no dramatic effect on *Bacillus* MSP dendrograms (Fig. 4a: LB and Fig. S6: LB, NA, BHI, PCA with gel views). *B. cereus*, *B. thuringiensis* and *B. mycoides* strains were still clustered in the *B. cereus* group with high similarity and all the tested strains in the *B. subtilis* group were firmly linked in the same clade. The MSP of other species like *B. megaterium* strains and *B. marisflavi* type strain were also consistently found in the same clades no matter which media were used. The high intensity peaks in MSPs are crucial in the identification and taxonomy of microorganisms. MSP gel view of type strains showed that high intensity peaks in each strain were not significantly different between the *Bacillus* strains incubated in the four different media (Fig. 4b).

### Range *m/z* 1000–3000 assisted the standard region (*m/z* 3000–15000) for differentiation of highly similar strains

**Figure 5:**
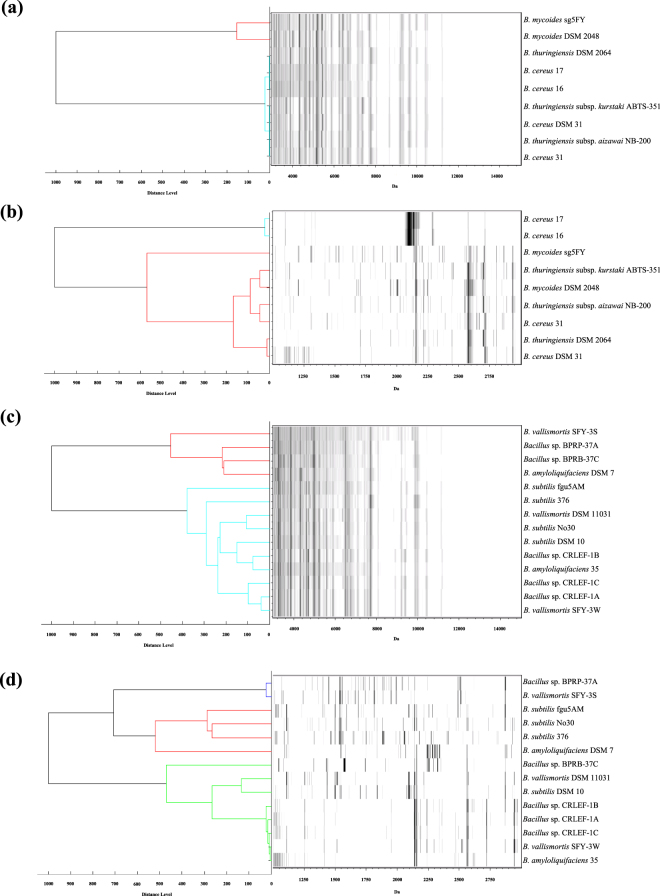
MSP dendrogram and spectra gel view of (**a, b**) *Bacillus cereus* and (**c, d**) *Bacillus subtilis* group species cultured on LB agar at 30 °C for 12 hours. The analysis and construction of dendrograms (**a**) and (**c**) are based on the *m/z* 3000–15000 range and dendrogram (**b**) and (**d**) are based on the *m/z* 1000–3000 range.

Many isolates in *B. subtilis* and *B. cereus* groups are not easily distinguished using single genomic approaches such as 16S rRNA sequencing or MALDI-TOF MS analysis in the range *m/z* 3000–15000. To investigate how to reinforce the differentiation of strains within these groups using MALDI-TOF MS, we set up the standard identification procedure (LB, 30 °C, 12 hours, *m/z* 3000–15000) and a modified procedure (LB, 30 °C, 12 hours, *m/z* 1000–3000) in this study. To eliminate the high intensity peaks in *m/z* 3000–15000 affecting distinguishability, *m/z* 1000–3000 was utilized in strain differentiation independently. The *B. subtilis* and *B. cereus* groups demonstrated much greater relative distance or different results for classification in MSP dendrogram analysis at *m/z* 1000–3000, in comparison to those derived from MSP at *m/z* 3000–15000 (Fig. 5).

## Discussion

The *B. subtilis* and *B. cereus* groups, which are highly genetically similar to each other, were the two targets of this study. Although, the strains were gathered from completely different origins, phylogenetic analysis based on 16S rRNA sequences in the species of each group showed no significant differences (Fig. 1). Some even showed 100% similarity in 16S rRNA sequences but had different colony morphologies, highlighting the difficulty of using 16S rRNA sequences to differentiate strains or species in these groups. Previously, 23S rRNA, *gyrB* gene and other pathogenic gene clusters have been used to support the classification and identification of these groups^22,23^; however, the results were either efficient or reliable, but not both, suggesting improvements are needed. MALDI-TOF MS-based microbial classification has also been used with some success to identify *Bacillus* pathogenic and non-pathogenic strains through the MSP^24^.

Protein profiles collected by MALDI-TOF MS have become an efficient method to rapidly identify microbial samples, especially for clinical diagnosis, and bacterial and fungal protein databases have been comprehensive for a decade^25^. Many species of *Bacillus* are collected in the commercial databases; however, because of endospores, which mix together with vegetative cells, classification and identification of *Bacillus* strains by using MALDI-TOF MS is particularly difficult. The variable vegetative-endospore cell composition directly affects identification of members of the *Bacillus* genus^13^. Moreover, the fraction of SASPs which is around 8% to 20% in different species of endospores is variable^26^, further hindering the accurate identification of several *Bacillus* species. Therefore, a method that can analyze total proteins of vegetative cells without endospore interference would be ideal for classification of endospore-forming bacteria. In this study, we were able to obtain endospore-free *Bacillus* through adopting simple culturing methods.

Optimizing the culture conditions is a straightforward way to avoid endospore formation. Growth rate directly influences the transformation from vegetative cells to endospores. Three culture conditions that affect the growth rate of *Bacillus*, temperature, incubation time and medium, were evaluated in this study. We demonstrated that temperature partially affected the MSP of some *Bacillus* strains. Of note, it had considerable effects on *B. subtilis* group at 30 °C and 37 °C within one day incubation (Fig. 2). Moreover, incubation periods of *Bacillus* on agar media also dramatically influenced the MSP and principal component analysis results of both the *B. cereus* group and the *B. subtilis* group, as well as other *Bacillus* strains (Fig. 3). Endospores were easily observed when *Bacillus* were mature or overgrown on agar media (Table S1). A previous study indicated that *B. subtilis* and *B. licheniformis* incubated for one, two and four days at 30 °C on PCA had no significant effect on protein profiles^27^. In this study, MALDI-TOF MS, endospore measurement and microscopy observation were used to confirm that some strains are affected to different extents depending on the growth rate of the *Bacillus*. The same phenomenon was reported by Chambers *et al*. who showed that endospores directly decreased the identification scores which were evaluated by MALDI-TOF MS, and disturbed identification of *B. subtilis* ^28^. Moreover, SASPs, the essential elements for endospore formation, were also detected in reference strains after 24 hours or longer after incubation, but not at 12 hours, and these results also verified that SASPs, including α-SASP, *β*-SASP and α/*β*-SASP, are usually observed under the standard conditions (LB, 24 hours, 37 °C)^24,29^. MSP dendrograms revealed how the vegetative-endospore cell mixture influenced the taxonomy of *Bacillus* and what conditions can be used to circumvent these situations (Fig. S5). Keeping the *Bacillus* sample as fresh as possible is a critical step to stabilize the result of identification and classification. Here we recommend the use of LB, 12 hours, 30 °C as standard culture conditions for *Bacillus* classification by MALDI-TOF MS.

The culture medium is one factor that has physiological effects on bacteria that further influences MALDI-TOF MS-based identification. Culture medium has been studied for the identification of many bacteria such as *E. coli* ^30^, *Yersinia* ^30^, *Lactobacillus* ^31^, *Staphylococcus* ^32^ *, Pseudomonas* ^32^ *, Salmonella* ^32^ *, Klebsiella* ^32^ and so on. Some results revealed highly significant differences when bacteria were cultured on different media. To confirm whether the culture media affect the MSP and classification, in this study, we chose four culture media, LB, NA, BHI and PCA, which are commonly used in isolation and incubation of *Bacillus*. In comparison with 16S rRNA phylogenetic analysis the MSP dendrograms demonstrated no significant differences (Figs 1 and 4). The results also showed no substantial change from one medium to another and the major peaks in mass spectra of all species had only inappreciable differences in intensity. Valentine *et al*. revealed that *B. subtilis* cultured on four different media showed slightly different spectral patterns; however, in our study *B. subtilis* were well identified no matter which culture medium was applied for MALDI-TOF MS analysis^30^ (Fig. 4 and Fig. S6).

In *Bacillus*, the major peptides and proteins over *m/z* 3000 are ribosomal subunit proteins, SASPs or other conserved domain and putative uncharacterized peptides and proteins that give protein fingerprints that are relatively consistent^13,33^. Nonetheless, analysis of mass signals in this range restricts the discrimination of strains, subspecies and even species like the *B. cereus* group and *B. subtilis* group. The mass region from *m/z* 1000–3000 is much more variable and it contains several important lipopeptide-like antibiotics, like surfactins, iturins and fengycins, which are synthesized by specific strains of *Bacillus* ^34^. Therefore, this region has the potential to distinguish the *Bacillus* strains from non-antibiotics producing strains through MALDI-TOF MS. In Fig. 5, the MSP dendrograms preprocessed at *m/z* 1000–3000 were compared with those at *m/z* 3000–15000. The non-canonical mass range showed an increase in distance between strains, and they also revealed different and diverse results of classification which can support classifying a number of isolates and eliminating duplicates in same origins. For example, in the *B. subtilis* group, *Bacillus* sp. SFY-3S and SFY-3W shared 100% similarity of 16S rRNA sequence but revealed quite a difference in this mass range. Although the biosynthetic gene expression of non-ribosomal peptides and antibiotics are manipulated by culture conditions, these results offer an alternative strategy for strain level differentiation or dereplication from numerous samples—known as subtyping, and can be efficient and reliable. Previously, a variation of this strategy was used with *Trichomonas vaginalis*, a parasitic protozoan. The analytical range was modified from *m/z* 3000–15000 to *m/z* 6000–10000 to exclude the non-relevant peaks of *T. vaginalis* and the identification of the protozoa was thus strengthened^35^.

In summary, here we confirmed that temperature and incubation period, which are critical to the formation of endospores, have considerable effects on *Bacillus* classification using MSP, regardless of the origin of the *Bacillus* samples. In addition, we found that culture media had an insignificant effect on MSP under fresh culture conditions (30 °C, 12 hours). Moreover, different mass detection ranges were able to support the classification of infraspecies. Culturing *Bacillus* with fresh culture conditions is important for classification by MALDI-TOF MS.

## Methods

### *Bacillus* strains and identification

A total of 30 strains collected from different sources were first identified as *Bacillus* by 16S rRNA. Seven strains were purchased from the Bioresource Collection and Research Center (BCRC), Taiwan. Two strains were collected from commercial bio-pesticides. The others were collected and isolated from terrestrial and marine biological material including plants and shrimps, and so on (Table 1). The 16S rRNA accession number of type strains and sequences of isolated strains are also shown in Table 1.

### Phylogenic analysis of *Bacillus* 16S rRNA sequence

The 16S rRNA sequences were amplified with 16S-8F primer [5′AGAGTTTGATCCTGGCTCAG3′] and 16S-1541R primer [5′AAGGAGGTGATCCAGCCGCA3′] by Expand High Fidelity PCR System (Roche Applied Science) following the manufacturer’s instructions^36^. The sequences were edited and aligned by MEGA 5.0^37^. The phylogenic analysis was constructed by MEGA 5.0 following the neighbor-joining method with 1000 bootstrap replicates.

### Culture conditions

The *Bacillus* in frozen stocks were recovered onto LB medium with 1.5% agar at 30 °C for at least 24 hours and single colonies were transferred to other media for further tests. For assay of incubation time, all *Bacillus* were cultured on LB 1.5% agar at 30 °C and single colonies were collected at 12, 24, 48 and 72 hours after inoculation. For assay of different culture temperatures, *Bacillus* was cultured on LB agar for 24 hours at 30 °C or 37 °C. For assay of different media, *Bacillus* were cultured on LB agar, NA, PCA and BHI agar for 12 hours at 30 °C.

### Counting of endospores

For the procedure, please refer to methods of Leuschner *et al*.^38^. Briefly, single colonies picked up from LB agar at 12, 24, 48 and 72 hours after inoculation were suspended in 100 μL sterilized water and 50 μL suspension was heated at 80 °C for 10 minutes and the rest was kept at room temperature. The samples were incubated on LB medium with 1.5% agar at 30 °C for at least 12 hours after properly diluting and spread onto agar plates. The total cell counts were obtained from untreated samples and endospore counts were from heated samples. The *Bacillus* samples, incubated for 72 hours and stained by Schaeffer and Fulton Spore Stain Kit (Sigma-Aldrich), were examined by Eclipse Ci-L microscope (Nikon) with eyepiece lens 10X, objective oil lens 100X and the software NIS Element D 4.5 (Nikon).

### Protein/peptide extraction for MALDI-TOF MS

The single colonies picked up from agar were suspended in the 300 μL sterile water and mixed with 900 μL absolute ethanol. The suspension was centrifuged at 17000 g for one minute then the supernatant was discarded. After the pellet was air-dried at room temperature, an appropriate amount (from 5 to 50 μL) of 70% formic acid was added and mixed with the pellet. The sample was added with equal amount of acetonitrile and then centrifuged at 17000 g for one minute to prepare the supernatant for MALDI-TOF MS analysis. The procedure was mainly derived from Sauer *et al*.^39^ and Shih *et al*.^40^.

### MALDI-TOF MS analysis

Peptide/protein extract (1 μL) was dropped on the MTP 384 target plate (Bruker Daltonics) and dried at room temperature. Then 1 μL of matrix solution (10 mg α-cyano-4-hydroxycinnamic acid in 1 mL standard solution: 50% acetonitrile and 2.5% trifluoroacetic acid) was dropped onto the target sample. The targets were detected by Autoflex Speed MALDI-TOF/TOF mass spectrometry (Bruker Daltonics) in linear positive mode in the range of *m/z* 1000–20000 (Detector Gain 8.1 × 2819 V) and the laser frequency was 500 Hz. The laser pulses of each target were 1500 laser shots from three 500 laser shots per position and five spectra were collected from five targets. The standards of calibration were Protein Calibration Standard for Mass Spectrometry (Bruker Daltonics) and the total protein extracted from *Escherichia coli* DH5α, and then the DH5α was used as a positive control of identification by Biotyper 3.1 (Bruker Daltonics) with scores higher than 2.3 against the DH5α MSP in the Biotyper database.

### Mass spectrum profile analysis

The raw mass spectra were preprocessed by Biotyoer 3.1 before further analysis according to Biotyper Preprocessing Standard Method (smoothing method: Savitski-Golay; baseline subtraction method: multipolygon; normalization method: maximum normalization). The range of mass spectra was restricted in *m/z* 3000–15000 (default mass range) or *m/z* 1000–3000 and the method of peak picking was the spectra classification method and the ratio of signal to baseline noise was 3. The maximum of the picked peaks in a spectrum was 100 and the minimum of desired peak frequency was 60%. The MSP dendrograms were generated by Biotyper MSP Dendrogram Creation Standard Method (distance measure: correlation; linkage method: average; score oriented). Principal component analysis was performed by ClinProTools 3.0. The raw spectra (*m/z* 3000–15000) were also prepared through default setting (smoothing method: Savitski-Golay; baseline subtraction method: top hat baseline) and the method of peak picking was the spectra classification method and the ratio of signal to baseline noise was 5. The scaling method of calculating the principal component analysis was level scaling. Each isolate with different incubation time had 5 spectra. The results of principal component analysis displayed data plotted according to the first three components (PC1, PC2 and PC3) which explained most of the variance in the data set.

## Electronic supplementary material

Supplementary Information
