# Supplementary material for: Bacillus Classification Based on Matrix-Assisted Laser Desorption Ionization Time-of-Flight Mass Spectrometry—Effects of Culture Conditions
Source: Sci Rep. 2017 Nov 14;7:15546. doi: 10.1038/s41598-017-15808-5 (PMC5686160; doi:10.1038/s41598-017-15808-5)
Supplement: Supplementary file 1 — Supplementary Information [file 41598_2017_15808_MOESM1_ESM.pdf]

Supplementary information

Bacillus Classification Based on Matrix-Assisted Laser Desorption Ionization Time-of-Flight Mass Spectrometry—Effects of Culture Conditions

Lin-Jie Shu and Yu-Liang Yang*

Agricultural Biotechnology Research Center, Academia Sinica, Taipei 11529, Taiwan

*Correspondence
Yu-Liang Yang: ylyang@gate.sinica.edu.tw

Table of Contents

**Figure S1**	**The colony morphology of *B. vallismortis* SFY-3S and *B. vallismortis* SFY-3W.**	**3**
**Table S1**	**The proportion of *Bacillus* endospore cell formation (vegetative cells/total cells) after 12, 24, 48, and 72 hours post incubation.**	**4**
**Figure S2**	**The micrographs of (a) *B. marisflavi* DSM 16204 and (b) *B. vallismortis* DSM 11031 incubated for 12 hours, 24 hours, 48 hours and 72 hours stained by Schaeffer-Fulton spore staining.**	**5**
**Figure S3**	**MSP dendrograms and spectral gel view of (a) *B. cereus* and (b) *B. subtilis* group species cultured for 12, 24, 48, 72 hours.**	**6**
**Figure S4**	**Three- and two-dimensional principal component analysis plots of *B. cereus* group, *B. subtilis* group, *B. megaterium*, *B. marisflavi* and other *Bacillus* species cultured for 12, 24, 48, 72 hours.**	**7**
**Figure S5**	**MSP dendrograms and gel-views of (a) *B. cereus* DSM 31 (b) *B. subtilis* DSM10 cultured for 12, 24, 48, 72 hours.**	**8**
**Figure S6**	**Biotyper analysis of *Bacillus* with different culture media.**	**9-12**

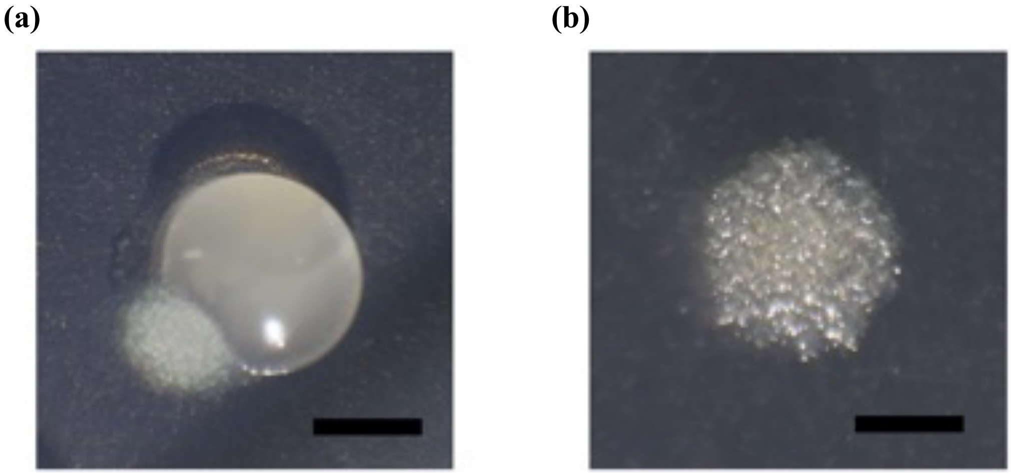

Figure S1. The colony morphology of (a) *B. vallismortis* SFY-3S and (b) *B. vallismortis* SFY-3W at 30°C for 24 hours on LB agar. Scale bars represent 2 mm.

Table S1: The proportion of *Bacillus* endospore cell formation (vegetative cells/total cells) after 12, 24, 48, and 72 hours post incubation.

Species	Endospore Fractions (%)			
	12-hour	24-hour	48-hour	72-hour
B. thuringiensis DSM 2046	0	0	0	0
B. mycoides DSM 2048	0	0	0	0
B. subtilis subsp. *subtilis* DSM 10	0	0.47	0.63	0.61
B. amyloliquefaciens DSM 7	0	0	10.00	41.33
B. cereus DSM 31	0	0	0	0.33
B. vallismortis DSM 11031	*	*	*	*
B. marisflavi DSM 16204	*	*	*	*
B. cereus 31	0	0.50	0.36	0.17
B. cereus 17	0	0	0.01	0.05
B. cereus 16	0	0	0	0.01
B. thuringiensis subsp. *kurstaki* ABTS-351	0	0.01	0.50	0.11
B. thuringiensis subsp. *aizawai* NB-200	0.1	0.01	4.40	5.14
B. mycoides sg5CY	0	0	0	0
B. subtilis 376	0	5	6.67	11.88
B. subtilis No30	0	0	0.16	0.14
B. subtilis fgu5AM	0	0.01	10.00	35.71
B. vallismortis SFY-3W	0	0.01	0.29	5.29
B. vallismortis SFY-3S	0	0.17	10.61	37.5
B. amyloliquefaciens 35	0	0.04	3.44	59.72
Bacillus sp. CRLEF-1A	0	1	7.5	78.05
Bacillus sp. CRLEF-1B	0	1.15	67.14	65.15
Bacillus sp. CRLEF-1C	0	0	33.33	97.96
Bacillus sp. BPRB37-A	0	0.13	11.67	89.47
Bacillus sp. BPRB37-C	0.07	0.91	6.07	95.56
B. licheniformis sp5BS	0	0	0.21	1.69
B. megaterium No56	0	0	0	0
B. megaterium 42	0	0	0	0
Bacillus sp. fgu5FY	0	0	0.01	0.03
B. safensis SC	0.08	2.96	0.02	0.23
Bacillus sp. UOY-1	0	0	0	0

* *B. vallismortis* DSM 11031 and *B. marisflavi* DSM 16204 did not present a fit to this endospore measurement method, substituted by light microscopic observation (Figure S2).

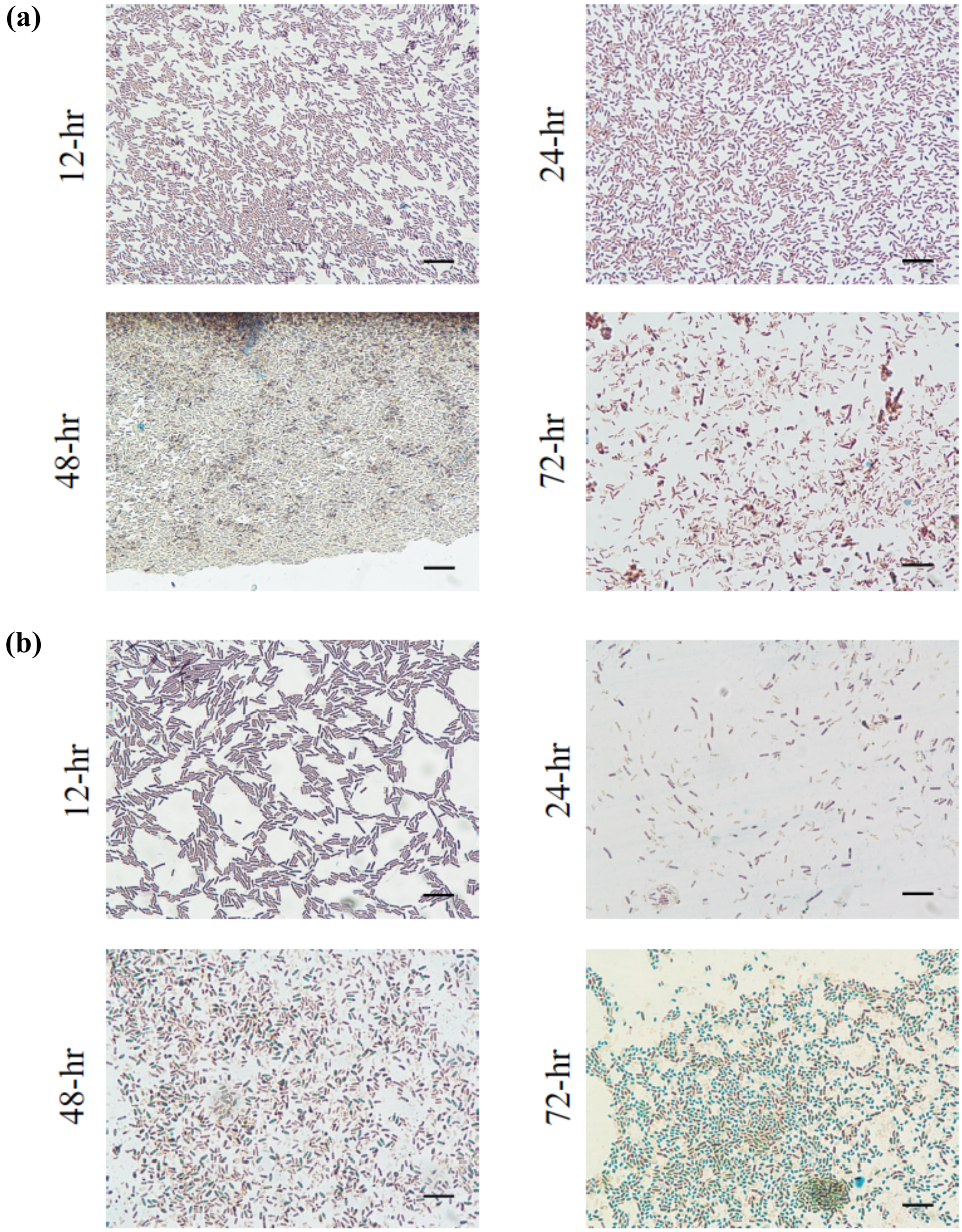

Figure S2. The micrographs of (a) *B. marisflavi* DSM 16204 and (b) *B. vallismortis* DSM 11031 incubated for 12 hours, 24 hours, 48 hours and 72 hours stained by Schaeffer-Fulton spore staining. They were cultured at 30°C on LB agar, and the samples were examined by Eclipse Ci-L microcope (Nikon) with eyepiece lens 10X, objective oil len 100X and software NIS Element D 4.5 (Nikon). Scale bars represent 10 μm.

(A)

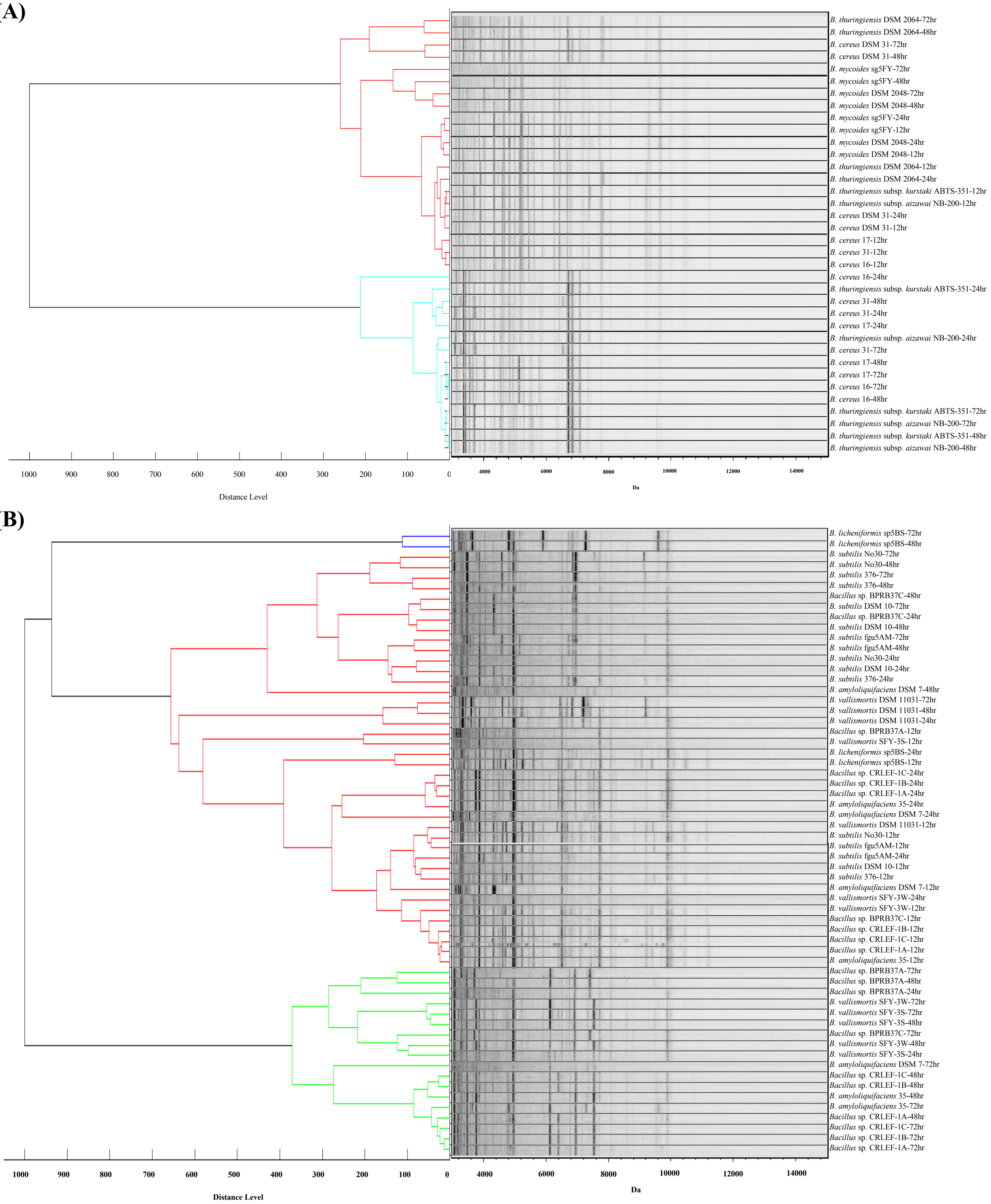

Figure S3. MSP dendrograms and spectral gel view of (a) *B. cereus* and (b) *B. subtilis* group species cultured for 12, 24, 48, 72 hours. Numbers behind the species and strain name indicate growth time.

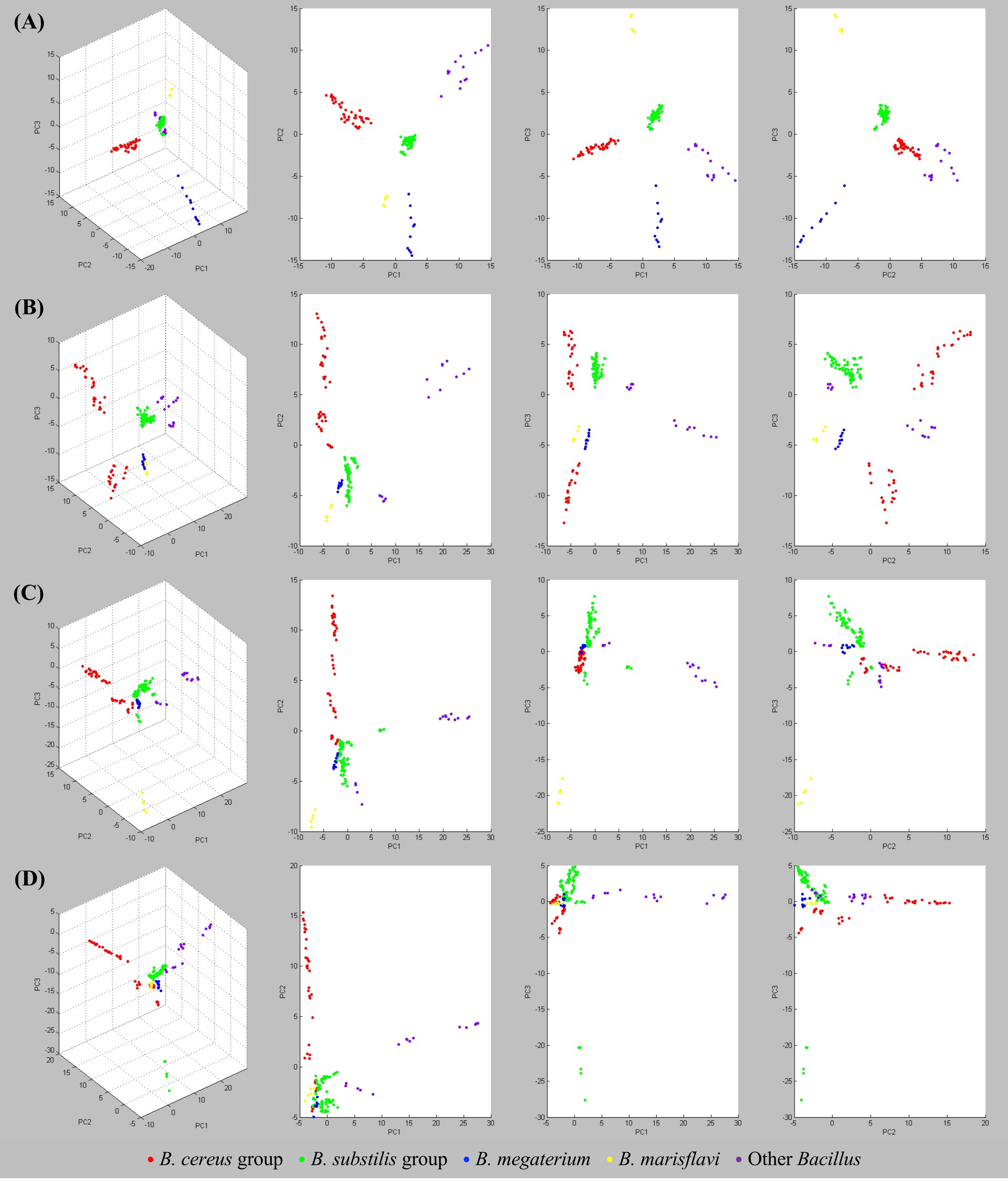

Figure S4. Three- and two-dimensional principal component analysis plots of *B. cereus* group, *B. subtilis* group, *B. megaterium*, *B. marisflavi* and other *Bacillus* species cultured for (A) 12 hours, (B) 24 hours, (C) 48 hours, and (D) 72 hours. The spot presents one spectrum and each *Bacillus* isolate includes five spectra. The plots were generated by first three principal components: PC1, PC2, and PC3.

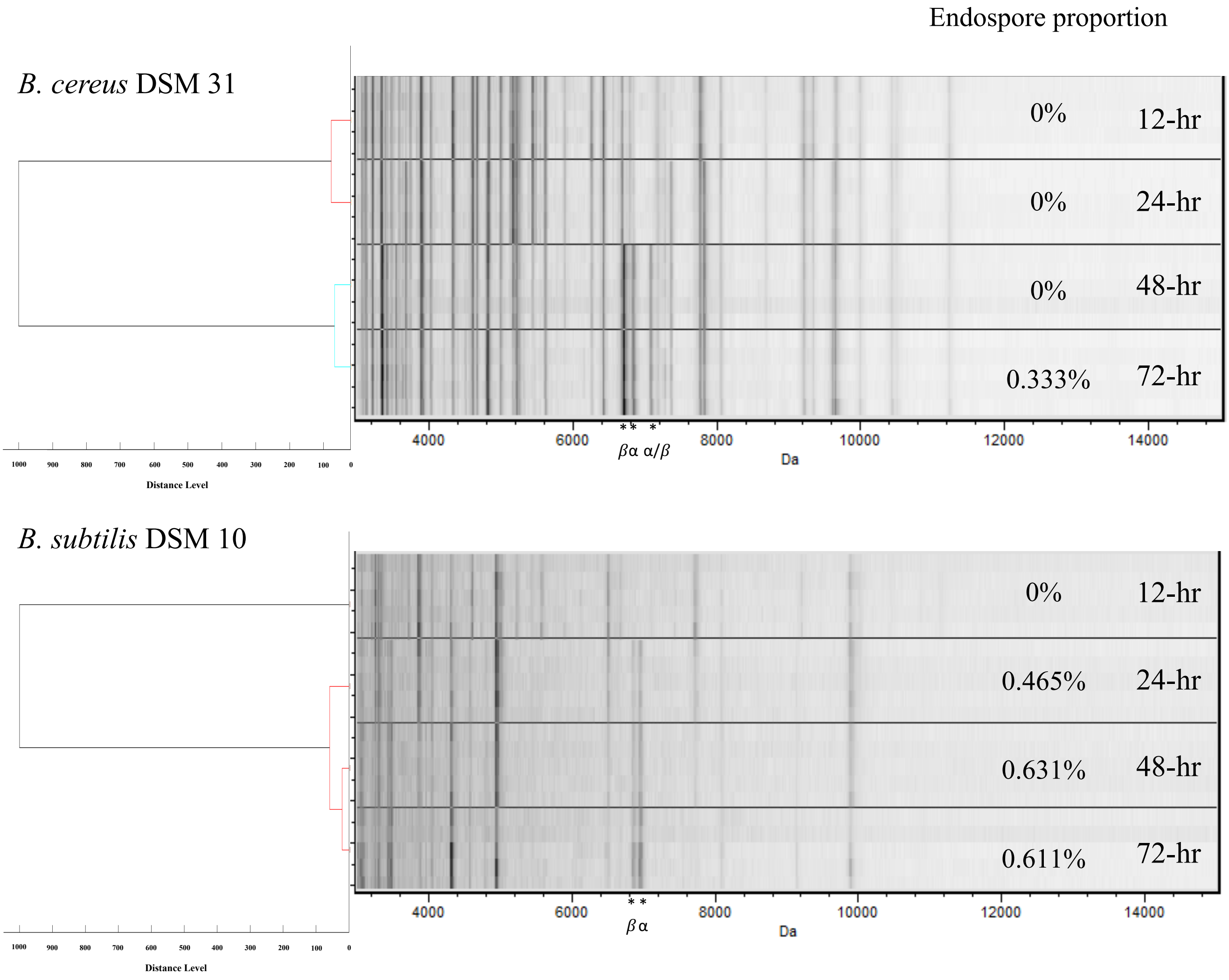

Figure S5. MSP dendrograms and gel-views of (a) *B. cereus* DSM 31 (b) *B. subtilis* DSM10 cultured for 12, 24, 48, 72 hours. The peaks of small acid-soluble proteins (SASPs) are indicated by asterisks. α, *β*, and α/*β* indicate the α-SASP, *β*-SASP, and α/*β*-SASP, respectively. Endospore proportions of samples are showed on the gel view, according to Table S1.

(a)

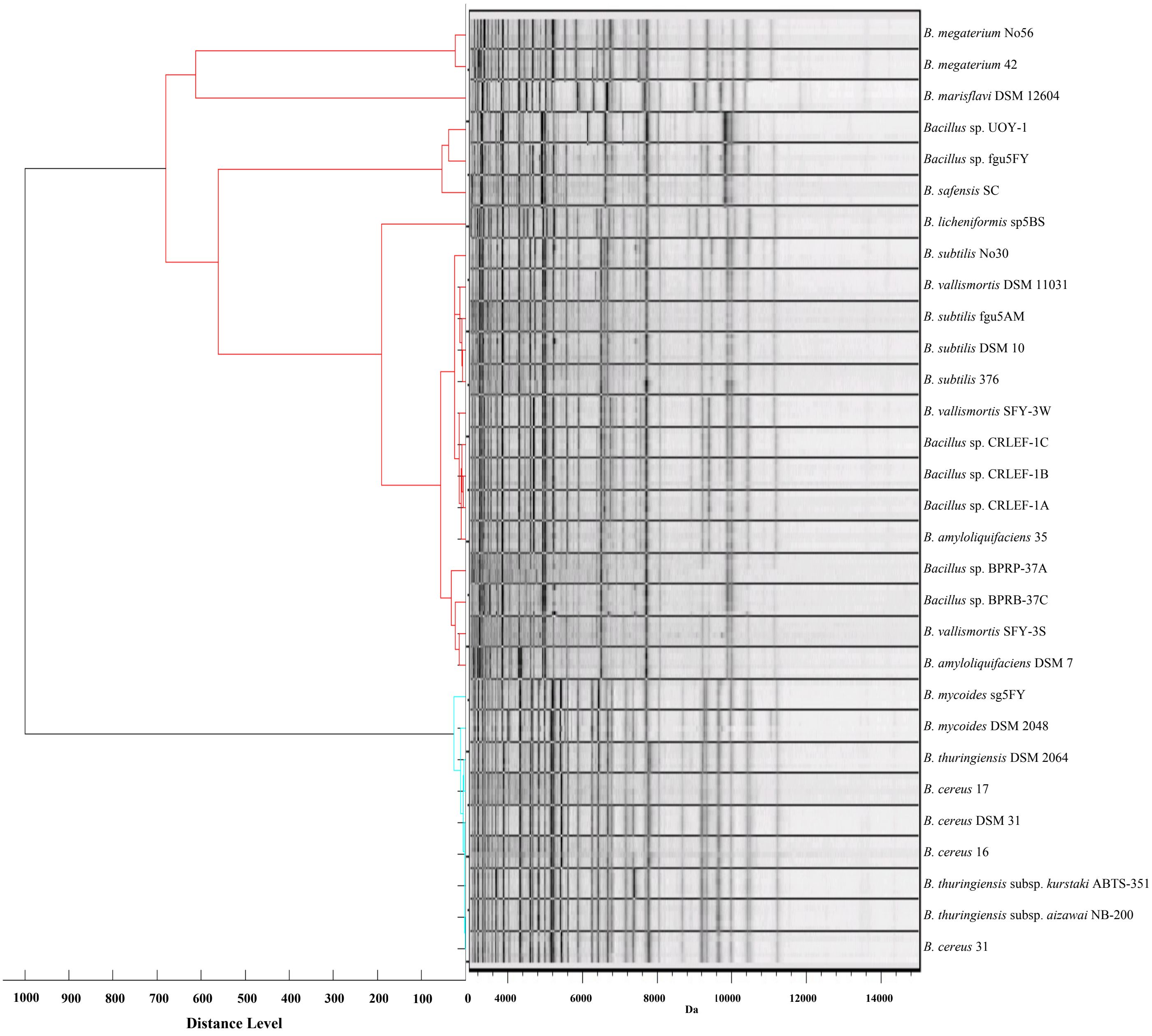

Figure S6. Biotyper analysis of *Bacillus* with different culture media. MSP dendrograms and spectral gel views of *Bacillus* cultured on (a) LB agar, (b) NA, (c) BHI agar and (d) PCA at 30°C for 12 hours. (Continued)

(b)

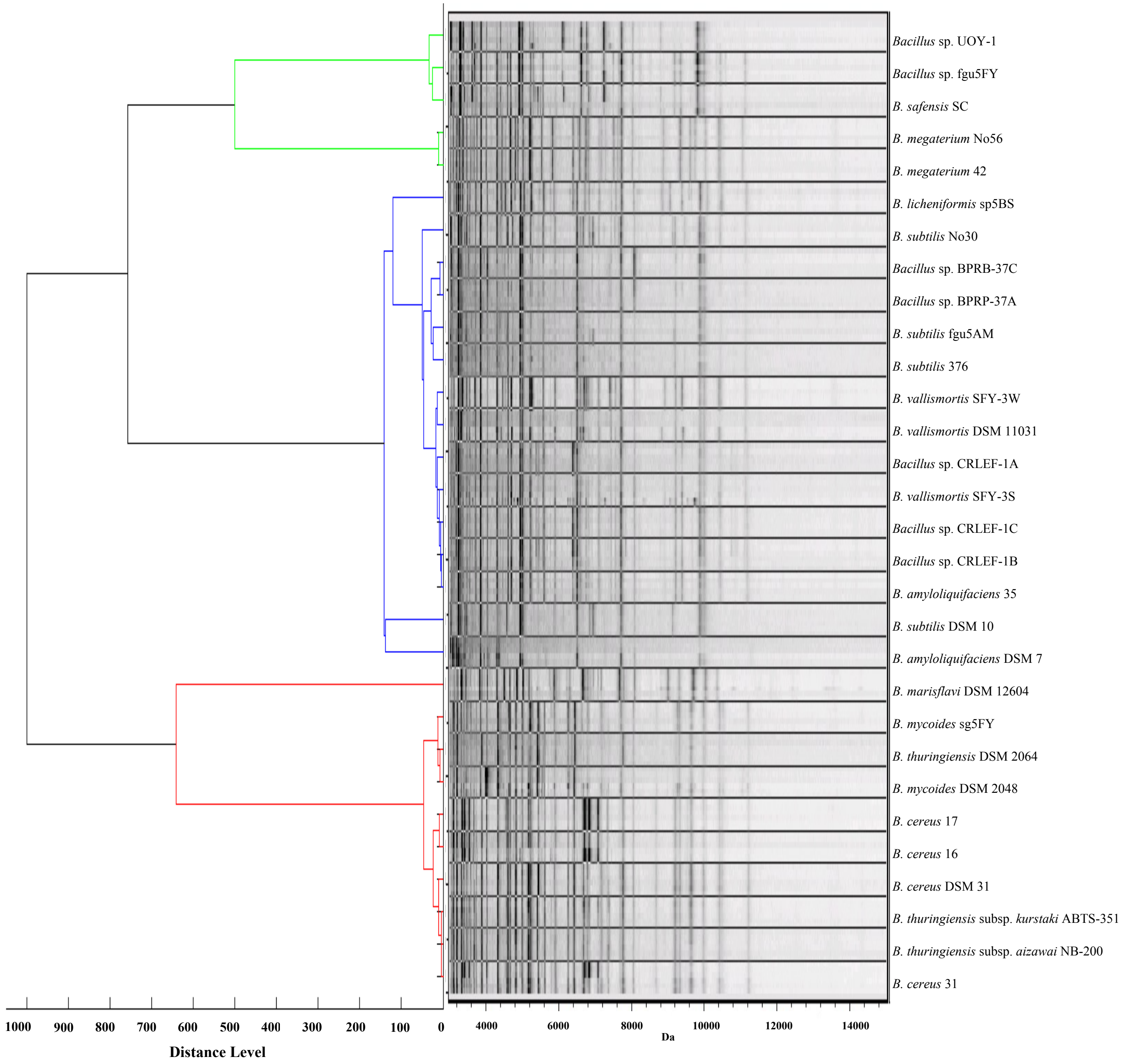

Figure S6. (Continued) Biotyper analysis of *Bacillus* with different culture media. MSP dendrograms and spectral gel views of *Bacillus* cultured on (a) LB agar, (b) NA, (c) BHI agar and (d) PCA at 30°C for 12 hours.

(c)

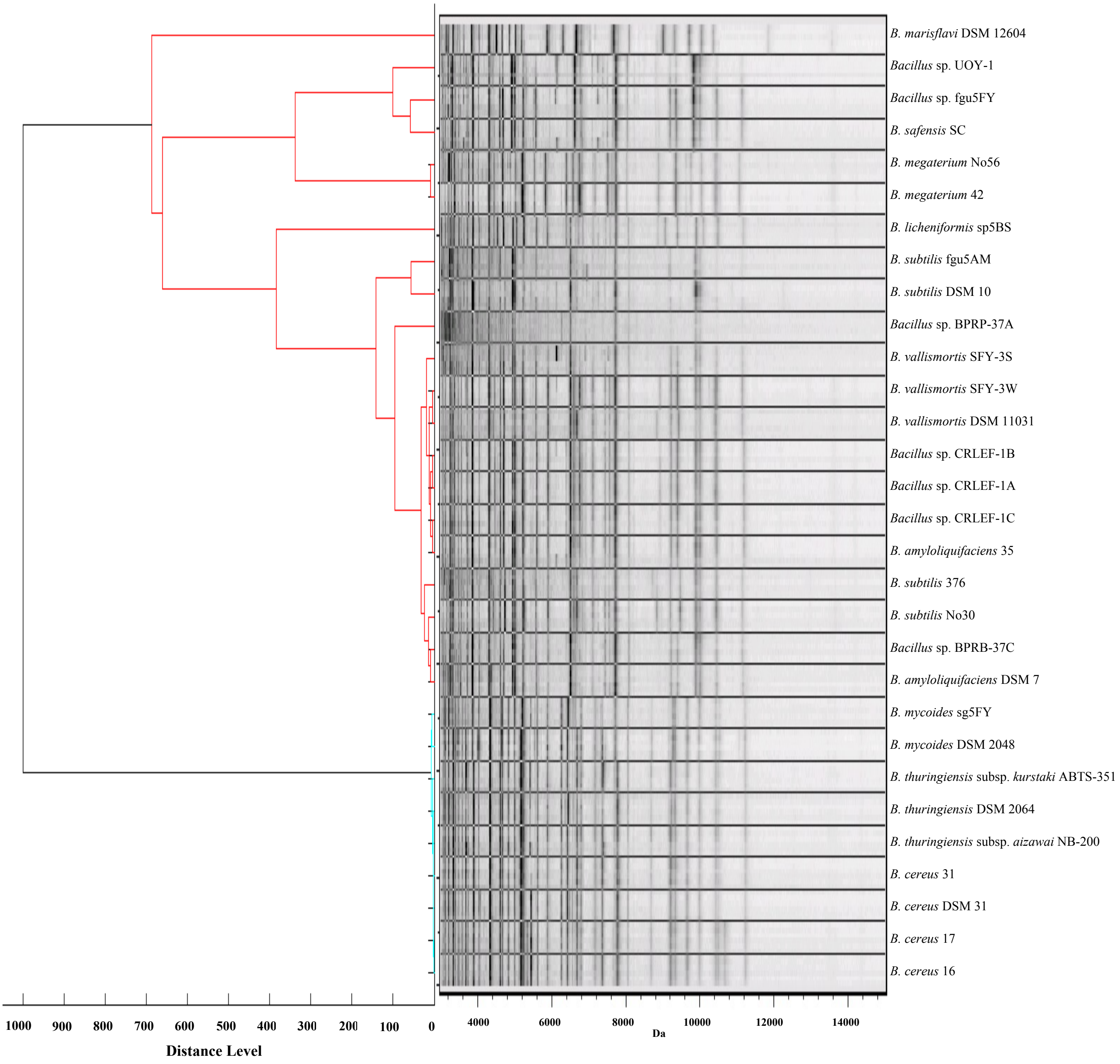

Figure S6. (Continued) Biotyper analysis of *Bacillus* with different culture media. MSP dendrograms and spectral gel views of *Bacillus* cultured on (a) LB agar, (b) NA, (c) BHI agar and (d) PCA at 30°C for 12 hours.

(d)

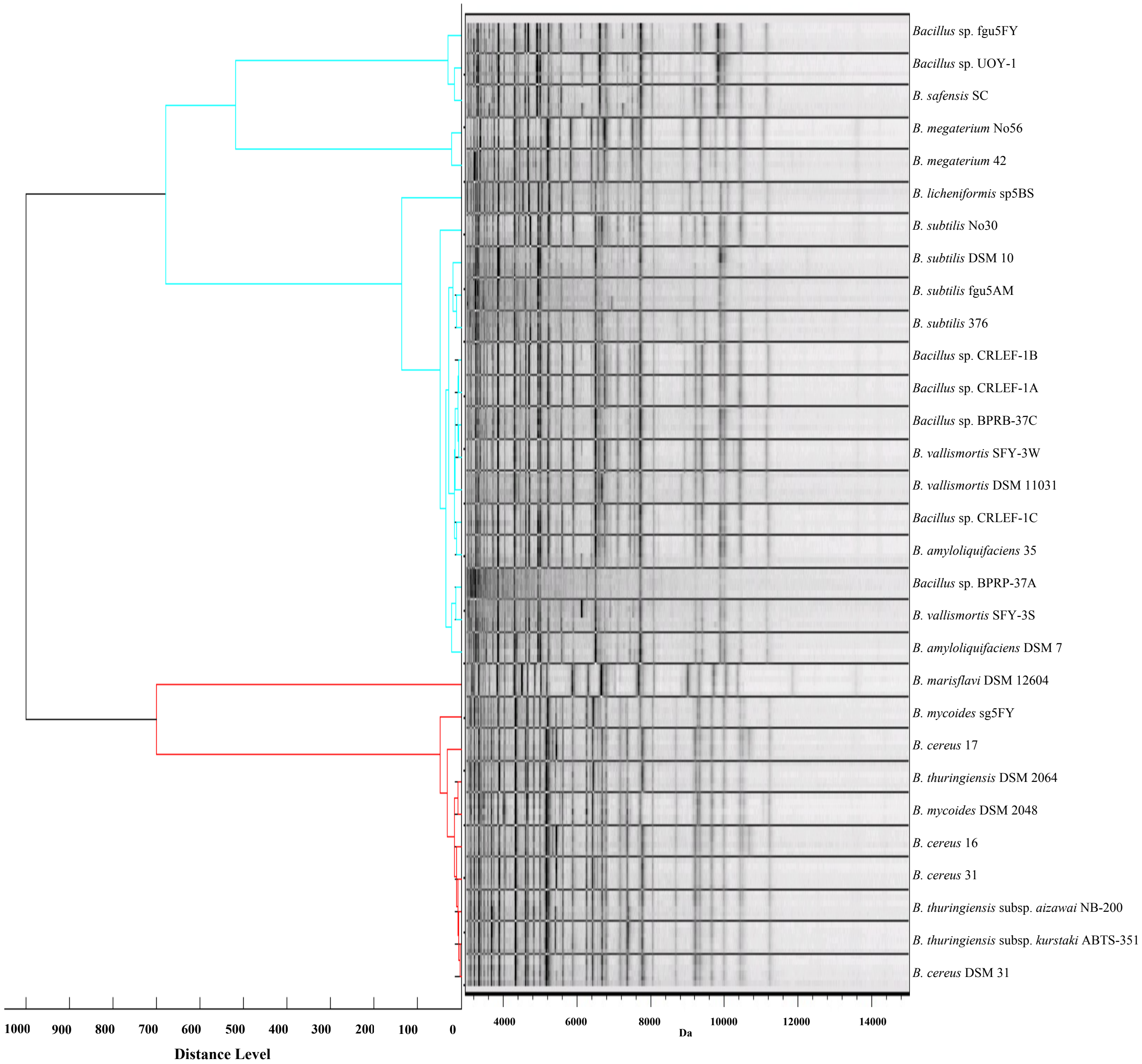

Figure S6. (Continued) Biotyper analysis of *Bacillus* with different culture media. MSP dendrograms and spectral gel views of *Bacillus* cultured on (a) LB agar, (b) NA, (c) BHI agar and (d) PCA at 30°C for 12 hours.
